# A dioxaborine cyanine dye as a photoluminescence probe for sensing carbon nanotubes

**DOI:** 10.3762/bjnano.7.190

**Published:** 2016-12-14

**Authors:** Mohammed Al Araimi, Petro Lutsyk, Anatoly Verbitsky, Yuri Piryatinski, Mykola Shandura, Aleksey Rozhin

**Affiliations:** 1 Nanotechnology Research Group, Aston Institute of Photonic Technologies, School of Engineering & Applied Science, Aston University, Aston Triangle, B4 7ET Birmingham, UK; 2 Engineering Department, Al Musanna College of Technology, Muladdah Musanna, P.O. Box 191, P.C. 314, Sultanate of Oman; 3 Institute of Physics, National Academy of Sciences of Ukraine, 46, prospekt Nauky, 03680 Kyiv, Ukraine; 4 Institute of Organic Chemistry, National Academy of Sciences of Ukraine, 5, Murmanska str., 02660 Kyiv, Ukraine

**Keywords:** dioxaborine cyanine dye, photoluminescence, resonant energy transfer, sensor, single-walled carbon nanotubes (SWNTs)

## Abstract

The unique properties of carbon nanotubes have made them the material of choice for many current and future industrial applications. As a consequence of the increasing development of nanotechnology, carbon nanotubes show potential threat to health and environment. Therefore, development of efficient method for detection of carbon nanotubes is required. In this work, we have studied the interaction of indopentamethinedioxaborine dye (DOB-719) and single-walled carbon nanotubes (SWNTs) using absorption and photoluminescence (PL) spectroscopy. In the mixture of the dye and the SWNTs we have revealed new optical features in the spectral range of the intrinsic excitation of the dye due to resonance energy transfer from DOB-719 to SWNTs. Specifically, we have observed an emergence of new PL peaks at the excitation wavelength of 735 nm and a redshift of the intrinsic PL peaks of SWNT emission (up to 40 nm) in the near-infrared range. The possible mechanism of the interaction between DOB-719 and SWNTs has been proposed. Thus, it can be concluded that DOB-719 dye has promising applications for designing efficient and tailorable optical probes for the detection of SWNTs.

## Introduction

Carbon nanotubes exhibit unique physical and chemical properties distinctive from other materials because of their extreme aspect ratio offering a number of exciting applications in reinforced plastics, conductive composites, sensors and photonic devices [1]. However, with the rapid growth of nanotechnology, carbon nanotubes pose a significant potential threat to health and environment in the near future due to their toxicity [1–5]. Therefore, efficient sensing techniques for nanotubes have to be developed before the harmful exposure of carbon nanotubes to ambient environment.

One of the most efficient and versatile techniques of chemical sensing is photoluminescence (PL) detection [6] involving a substantial increase of the PL emission due to the presence of target molecules, i.e., carbon nanotubes. The amplification of the PL signal from single-walled carbon nanotubes (SWNTs) can be obtained via covalent formation of sp^3^-defects on the tube surface [7], encapsulation of small organic molecules inside the SWNTs [8] and non-covalent interaction of the tube sidewall with π-conjugated organic compounds [9–12]. Considering sensors, particular attention has to be paid to the PL enhancement in aqueous media, like the complexation and resonant energy transfer (RET) from cyanine dyes to the SWNTs covered by anionic surfactants in water [12]. However, a drawback of these cyanine-based systems is the RET in the range of PL excitation wavelengths, where the SWNTs have substantial intrinsic emission. As a result of the RET, PL intensity is amplified relatively to the intrinsic emission of SWNTs, so the complexation effect has limited relative sensitivity.

**Figure 1:**
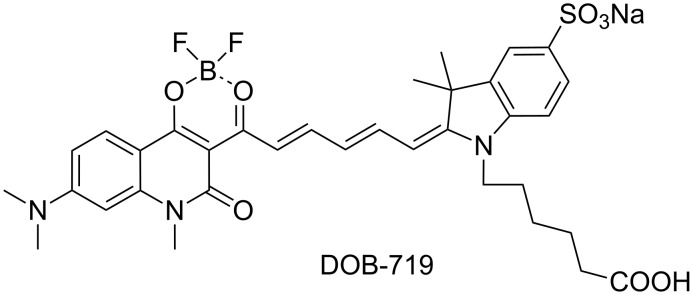
The chemical structure of DOB-719.

In this paper, we focused on studies of an interaction of SWNTs with the indopentamethinedioxaborine dye (DOB-719) having an extended π-conjugated system (Figure 1) and absorption in near infrared (NIR) range. The choice of the dye is driven by its unique interaction with the SWNTs resulting in the emergence of new PL peaks in the range of excitation wavelength at 650–780 nm, where the PL emission of the SWNTs is very low. Generally, polymethine dyes with a terminal dioxaborine group have intense PL signals, efficient two-photon absorption, high hyperpolarizability [13], and features of effective PL probes for amines and ammonia [14–16]. Optical properties (absorption and PL) of monomeric and dimeric forms of DOB-719 were reported in [16] showing that there is a weak interaction of SWNTs with DOB-719. The novelty of the present study is an elucidation of the interaction of the dye with the SWNTs providing clear evidences of RET from DOB-719 to SWNTs. The new results obtained in these studies allow us to propose the mechanism of interaction between the SWNT and DOB-719, where the dye is attracted to the SWNT surface via π–π stacking with the hydrophobic part while the hydrophilic groups are facing the aqueous medium.

## Results and Discussion

**Figure 2:**
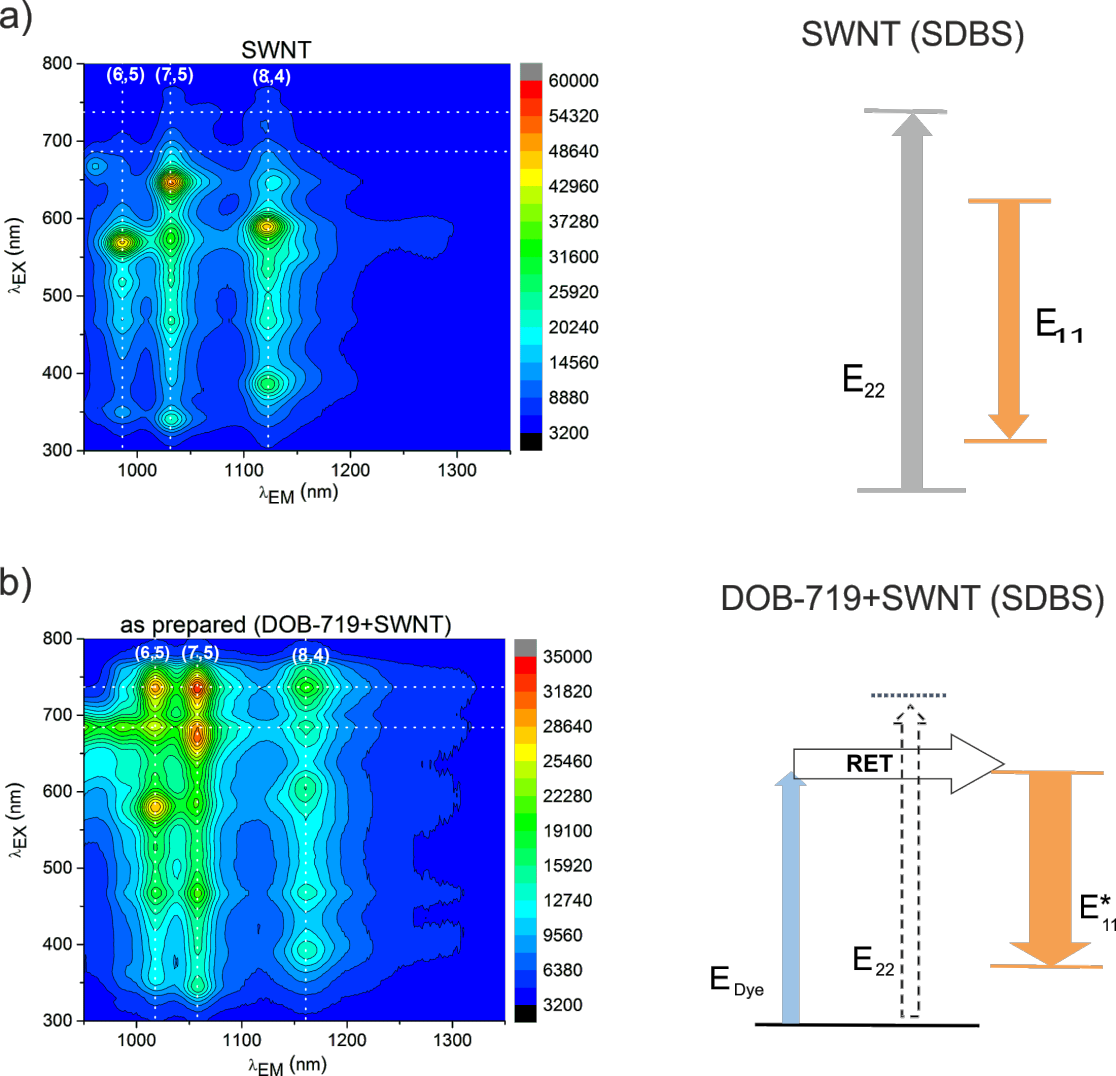
PLE maps (left) and the energy diagrams (right) for (a) dispersion of neat SWNTs and (b) as-prepared mixture of DOB-719 with the SWNT in water. Vertical dashed lines indicate λ_EM_ positions of E_11_ for SWNT of (6,5), (7,5), and (8,4) chirality, horizontal dashed lines indicate the position of new PL peaks at λ_EX_ = 735 nm (the RET from DOB-719 to the SWNT) and the position of DOB-719 monomers maximum at λ_EX_ = 685 nm. High PL intensities are coded in red colour, whereas low intensities are coded in blue colour. The concentration of DOB-719 in panel (b) is 0.001 mg/mL.

We have studied the interaction of DOB-719 with SWNTs by spectral analysis of absorption and PL for as-prepared and aged (measured 24 h after the mixing) mixtures of DOB-719 with SWNTs in water. Essentially, an admixture of the dye (0.001 mg/mL) to SWNTs dispersed with sodium dodecylbenzenesulfonate (SDBS) resulted in the emergence of several new spectroscopic features compared to neat dye and neat SWNTs. Figure 2 shows the PL excitation–emission (PLE) maps, with the *X*-axis representing the emission wavelengths (λ_EM_) and the *Y*-axis representing the excitation wavelengths (λ_EX_) for neat SWNTs (Figure 2a) and as-prepared mixtures of DOB-719 with SWNTs (Figure 2b). In Figure 2a, the predominant (6,5), (7,5), and (8,4) chiralities of the SWNTs are clearly seen in the range of λ_EX_ at E_22_, E_33_, and E_44_ (300–800 nm) and λ_EM_ at E_11_ (950–1350 nm) [17–18]. In Figure 2b, the same PL features of the predominant SWNT chiralities are supplemented with new peaks in the range of λ_EX_ = 650–780 nm (with maxima at λ_EX_ = 735 nm) and λ_EM_ corresponding to the E_11_ SWNT emission levels. The new features in the PLE maps for the mixtures of DOB-719 with the SWNTs appear because of the formation of a nanostructured complex of the dye with the SWNT. A similar complexation phenomenon has been demonstrated recently for systems consisting of SWNTs and the cyanine dye astraphloxin [12,19]. However, the present system is different (not only because of the emergence of new PL peaks in the range of λ_EX_ = 650–780 nm) because of a strong redshift of the intrinsic SWNT peaks in the λ_EM_ (Table 1) and the quenching of these SWNT emission peaks. The strong redshifts for the λ_EM_ maxima of the mixtures in comparison with the neat nanotubes for all chiralities are summarised in Table 1. The variations of redshifts for different SWNT chiralities can be explained by a structural matching of the π-electron systems of SWNTs with particular chirality and the developed π-electron system of DOB-719. The different redshifts provide not only sensitivity, but also selectivity of the PL detection towards the nanotube diameters.

**Table 1:** Peaks of intrinsic PL emission (E_11_) for the neat SWNT dispersions and the as-prepared mixtures of DOB-719 with SWNTs, redshifts of E_11_ in PL due to an admixture of the dye.

	λ_EM_ (E_11_ or E_11_*)		
	
SWNT chirality	dispersion of the SWNT	as-prepared mixture of DOB-719 and the SWNT	redshift

(6,5) at λ_EX_ = 570 nm	985 nm (1.258 eV)	1018 nm (1.217 eV)	33 nm (41 meV)
(7,5) at λ_EX_ = 650 nm	1032 nm (1.201 eV)	1057 nm (1.172 eV)	25 nm (29 meV)
(8,4) at λ_EX_ = 590 nm	1121 nm (1.105 eV)	1161 nm (1.067 eV)	40 nm (38 meV)

The energy diagram shown at the right-hand side of Figure 2a represents a two-component system consisting of the anionic surfactant SDBS and the SWNTs in water, where the anionic surfactant forms micelles around the nanotubes having typical exciton energy levels of PL excitation at E_22_ and emission at E_11_. In Figure 2b, the three-component system is modelled, where DOB-719 is attached to the SWNT surface via π–π stacking of the hydrophobic parts (π-conjugated frame) while the aqueous medium is faced by the hydrophilic parts (SO_3_^−^ and COO^−^). The non-covalent attachment of the dye to the nanotube results in the efficient RET at the excitation wavelength in the range of 700–760 nm and a strong redshift of the E_11_ levels (to E*_11_) in the NIR range (Table 1). The excitation energy of DOB-719 in this range (*E*_Dye_) is transferred to the SWNT levels (E*_11_) via the RET. This way, DOB-719 (donor of energy) and SWNT (acceptor of energy) form nanostructured complexes via non-covalent interaction. As a result, the diagrams model the emergence of new PL peaks due to formation of non-covalent complexes of DOB-719 with the SWNT. The complexes of such dyes have high potential to be used as an effective PL probe for the detection of carbon nanotubes considering that there is clear dependence of the RET response on the SWNT concentration (see Supporting Information File 1, Figure S1).

**Figure 3:**
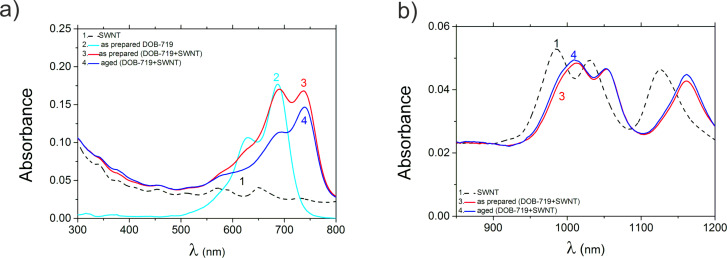
Absorption spectra in (a) the visible and (b) the NIR range for dispersions of SWNT (1), aqueous solutions of neat DOB-719 (2), as-prepared mixtures of DOB-719 with SWNT (3), and aged mixtures of DOB-719 with SWNT (4). The concentration of DOB-719 is 0.001 mg/mL.

Figure 3 shows the absorption spectra for the mixture of DOB-719 with the SWNT in comparison with its components. To understand the nature of the new PL features (in the range of λ_EX_ = 650–780 nm) we have estimated the absorption peak positions using Gaussian deconvolution. The absorption of the neat SWNT in the range of 300–800 nm (Figure 3a, curve 1) exhibits low intensity E_22_ and E_33_ excitonic transitions [20]. The neat DOB-719 (Figure 3a, curve 2) has a maximum absorption at 687 nm (1.80 eV) featuring monomers (free molecules) of the dye in aqueous solution [16]. The second peak has vibrational (631 nm; 1.96 eV) and dimeric (623 nm; 1.99 eV) components for DOB-719 molecules as shown and discussed in Figure S2 (Supporting Information File 1). The absorption spectrum of as-prepared mixture of DOB-719 with SWNTs (Figure 3a, curve 3) has two maxima at 692 nm (1.79 eV) and 742 nm (1.67 eV), where the former peak is a signature of the dye monomers. The latter peak correspond to the dye molecules associated with the SWNTs and is redshifted by 50 nm comparing to the absorption maximum of monomeric peak [12]. The intensity ratio of these peaks allows us to monitor both the amount of the dye monomers in the mixture and the amount of the dye associated with the SWNTs. Roquelet et al. reported a similar behavior for SWNT–porphyrin complexes, where an overlapping 20 nm split in the porphyrin (Soret) band was observed [21]. Here, the emergence of the 50 nm redshifted peak in the mixtures hinders a strong overlap with the monomer absorption band. The absorption spectrum of the aged mixture (measured 24 h after mixing, Figure 3a, curve 4) has only one maximum at 742 nm (1.67 eV) and a short wavelength shoulder (peak after Gaussian deconvolution at 700 nm (1.77 eV)). Thus, the relative contribution of absorption for the dye–SWNT complexes in the aged mixtures is much higher comparing to the as-prepared mixture.

The two-component mixture of SDBS and DOB-719 was studied resulting in no significant change of the absorption (Supporting Information File 1, Figure S3, curve 3) and PL spectra of the dye in the presence of SDBS. There is no evidence for the interaction between the surfactant (at premicellar and micellar concentrations) and the dye. Referring to our previous studies on the interaction of SDBS and astraphloxin (a polymethine dye with cyanines at both terminal groups) resulting in the aggregation of the dye. There is no such behaviour in the case of DOB-719.

In addition, we have studied the three-component system of DOB-719, SDBS, and SWNTs, where an extra amount of surfactant was added (see Supporting Information File 1, Figures S3–S5) to obtain a concentration of SDBS above the critical micellar concentration of 0.15 mg/mL [22]. The three-component mixture at micellar concentration of SDBS does not exhibit new bands in the range of 700–780 nm (in absorption and PL excitation). Thus, no RET from DOB-719 is possible when the micelles of SDBS fully cover the SWNTs. This shows another dissimilarity of the studied system in comparison with SWNT–SDBS–astraphloxin, where the RET was equally efficient at micellar concentrations of SDBS [19].

The absorption of the neat SWNTs in the range of 850–1200 nm exhibits E_11_ excitonic peaks [20] at λ = 986, 1031, and 1127 nm (Table 2; Figure 3b, curve 1). As a result of the dye admixture to the SWNTs, the E_11_ peaks are red-shifted in comparison to neat SWNTs (Table 2, Figure 3b). However, the E_11_ peaks of the mixtures practically do not change due to the aging (Figure 3b, curves 3 and 4). The E*_ii_* optical transition energies of the SWNTs strongly depend on the dielectric constant of both the nanotubes and the background of their surroundings (ε_bg_). Dielectric-screening effects become particularly evident when the SWNTs dispersed in liquids are compared to the nanotubes in air [23], so an increase of ε_bg_ results in a redshift of the E*_ii_* transition energies [23–25]. On one hand, in our work, the maximum and the minimum shifts in E_11_ exciton energy are 32 and 25 meV, respectively (Table 2), which are lower than the average shifts of E_11_ = 55 meV obtained in [23]. On the other hand, the redshifts in Table 2 are much lower than the redshift (of a few millielectronvolts) achieved when the surfactant around the nanotubes is diluted [25]. In the complex of DOB-719 with SWNT, the local increase of ε_bg_ can result from (i) a better access of water to the SWNTs and/or (ii) polar groups of the dye getting in close proximity to the nanotube surface. Thus, the nanotube transitions at E_11_ show significant redshifts, which are attributed to increase of ε_bg_ around the nanotubes due to the presence of the dye molecules.

**Table 2:** Peaks of absorption (E_11_) for the neat SWNT dispersions and the as prepared mixtures of DOB-719 with the SWNT, redshifts of E_11_ in absorption due to an admixture of the dye.

predominant chirality of E_11_ peaks [17]	dispersion of the SWNTs	as-prepared mixture of DOB-719 and the SWNT	redshift

(6,5)	986 nm (1.257 eV)	1011 nm (1.225 eV)	25 nm (32 meV)
(7,5)	1031 nm (1.202 eV)	1053 nm (1.177 eV)	22 nm (25 meV)
(8,4)	1127 nm (1.100 eV)	1161 nm (1.068 eV)	34 nm (32 meV)

**Figure 4:**
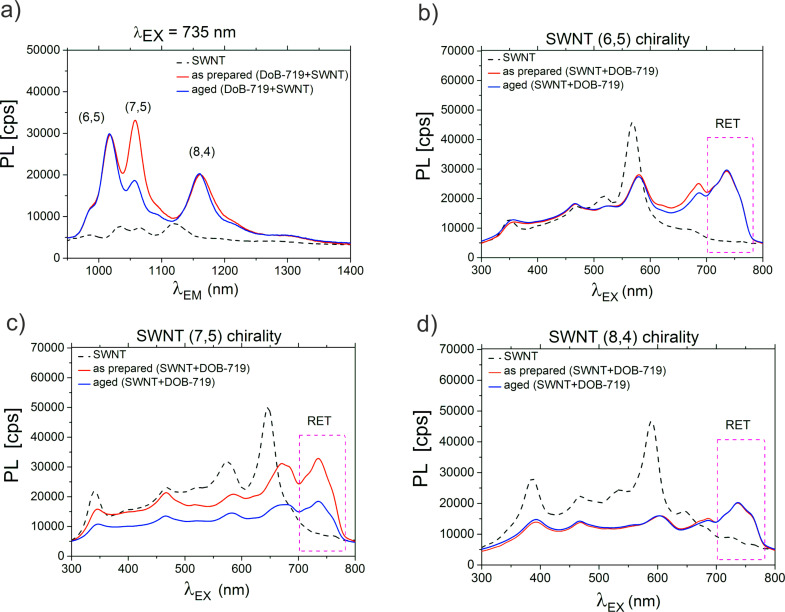
PL spectra for the as-prepared (red curves) and aged (blue curves) mixtures of DOB-719 and SWNTs and dispersions of neat SWNTs (dashed curves) in water. (a) PL emission spectra at λ_EX_ = 735 nm and PL excitation spectra (b) (6,5), (c) (7,5), and (d) (8,4) chiralities. PL spectra for the as-prepared and aged mixtures are extracted at λ_EM_ = 1016, 1059, and 1161 nm for the (6,5), (7,5), and (8,4) chiralities, respectively. PL spectra for neat SWNTs are extracted at λ_EM_ = 985, 1032, and 1121 nm for the (6,5), (7,5), and (8,4), respectively. The RET from the dye to the SWNT at λ_EX_ = 735 nm is indicated in panels b–d.

To provide better insight on the new spectral features in Figure 2 and Figure 3, we extracted the PL emission and excitation spectra for the as-prepared and aged mixtures from the PLE maps (Figure 2 and Figure S6, Supporting Information File 1). The PL emission spectra (Figure 4a) were extracted at λ_EX_ = 735 nm attributed to the complexation of DOB-719 with the SWNTs. The excitation spectra (Figure 4b–d) for the as-prepared and aged mixtures were extracted at λ_EX_ = 1016, 1059, and 1161 nm corresponding to the (6,5), (7,5), and (8,4) chiralities, respectively. For neat SWNTs the excitation spectra were extracted at λ_EX_ = 985, 1032, and 1121 nm referring to the (6,5), (7,5), and (8,4) chiralities, respectively. Importantly, the PL intensities for the (6,5) and (7,5) chiralities at λ_EX_ = 735 nm have increased (approximately five-fold) for the as-prepared mixtures comparing those of neat SWNTs. However, the PL intensity for the (8,4) chirality at λ_EX_ = 735 nm has grown approximately 2.5-times for the as prepared mixtures. This indicates that the RET from the dye attached to the (6,5) and (7,5) chiralities SWNT is more efficient than the RET for the (8,4) chirality. Thus, we can well discriminate the excitation peaks from excitonic levels of SWNTs and the RET from the dye complexed to the SWNT, where the RET has maximum at λ_EX_ = 735 nm. Finally, the ageing of the mixtures affects severely the complexes formed with SWNTs of (7,5) chirality (Figure 4a,c), whereas the complexes of (6,5) and (8,4) chirality are quite stable.

In Figure 2 and Figure 4, one can notice that, as a result of admixing DOB-719 to the SWNTs, the PL intensity of the intrinsic SWNT emission was quenched significantly for all chiralities (ca. 40% at λ_EX_ = 570 nm, λ_EM_ = 985 nm for (6,5); ca. 40% at λ_EX_ = 650 nm, λ_EM_ = 1032 nm for (7,5); ca. 70% at λ_EX_ = 590 nm, λ_EM_ = 1121 nm for (8,4)). This finding indicates that the interaction of DOB-719 with the SWNTs alters the intrinsic PL properties of the nanotubes. However, in the complexation system of astraphloxin–SWNT, intrinsic PL peaks of the SWNTs remained unchanged [12]. The moderate quenching of intrinsic SWNT emission evidences direct interaction of DOB-719 and the SWNT, which in case of the astraphloxin-based systems is restricted by the presence of the surfactant. Assumingly, SDBS does not affect the interaction of DOB-719 and the SWNT.

**Figure 5:**
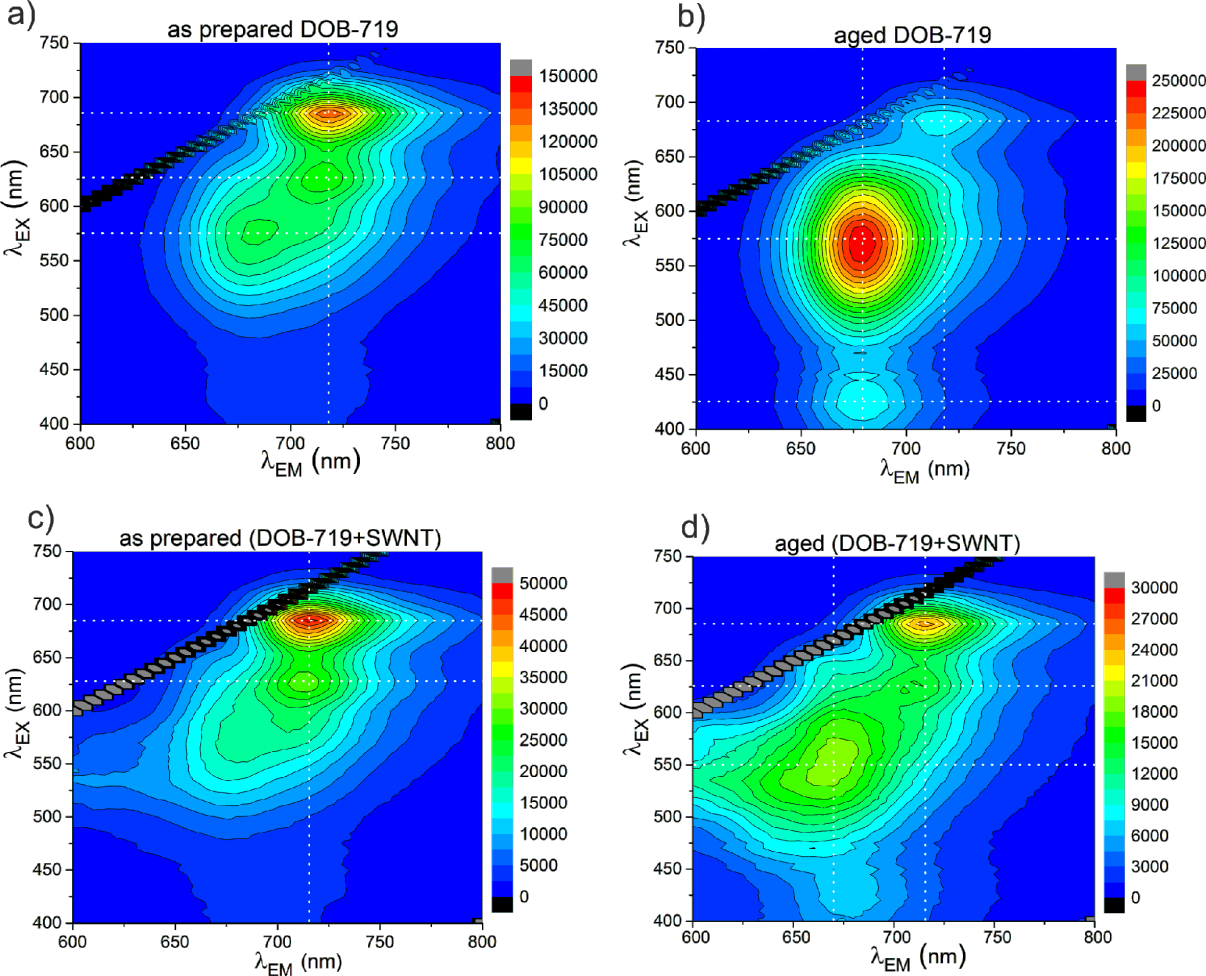
PLE maps of aqueous solutions for (a) the as-prepared and (b) aged neat DOB-719 as well as (c) the as-prepared and (d) aged mixtures of DOB-719 with SWNTs in water. High PL intensities are coded in red colour, whereas low intensities are coded in blue colour. The concentration of DOB-719 is 0.001 mg/mL.

To understand the nature of the changes in the mixtures, the PLE maps in the emission range of the dye were analysed (Figure 5). We have put together the PLE maps for the as-prepared and aged samples of both neat DOB-719 (Figure 5a,b) and the mixture of DOB-719 with the SWNT (Figure 5c,d) registered in the range of λ_EX_ = 400–750 nm and λ_EM_ = 600–800 nm. The PL peak with λ_EX_ = 685 nm and λ_EM_ = 720 nm (Figure 5) correlates with the monomeric peak in the absorption spectra at 687 nm (Figure 3a and Supporting Information File 1, Figure S2). The second band with weak PL in the range of λ_EX_ = 615–640 nm and λ_EM_ = 700–725 nm (in Figure 5a,c,d) could be related to both the dimeric PL of DOB-719 (λ_abs_ = 623 nm) and/or a second vibrational transition of the monomers (λ_abs_ = 631 nm) discussed in Supporting Information File 1 in Figure S2. In Figure 5b, the PL intensity of the monomeric peak (with λ_EX_ = 685 nm and λ_EM_ = 720 nm) for aged neat DOB-719 is quenched (to approx. a half) and a new band with strong PL appears. This band has a maximum at λ_EX_ = 575 nm and λ_EM_ = 680 nm, and its intensity is about four times higher than that of the as-prepared sample. In this spectral range, the as-prepared dye has only a weak and featureless shoulder (Figure 5a). The above new band and the low-intensity band in the range of λ_EX_ = 400–450 nm and λ_EM_ = 660–700 nm (Figure 5b) correspond to the new bands of absorption developed with time at λ= 335–600 nm (Supporting Information File 1, Figure S7, curves 2 and 3). These new features in the aged samples can be associated with the product of the dioxaborine cycle hydrolysis [13].

The PLE maps in the dye emission range for the mixtures show that the PL intensity of the monomeric peak (at λ_EX_ = 685 nm and λ_EM_ = 720 nm) is quenched down to about a third for both the as-prepared (Figure 5a,c) and the aged (Figure 5b,d) mixtures. The quenching of the monomeric peak due to the ageing is almost the same (down to about a half) for the neat dye (Figure 5a,b) and for the mixtures with the SWNT (Figure 5c,d). Importantly, the PL intensity of the band at λ_EX_ = 575 nm and λ_EM_ = 680 nm (resulted from the hydrolysis of dioxaborine cycle) does not change significantly after ageing of the mixture (Figure 5c,d). As it has been discussed before, in the neat dye the same band grew about four-fold during the ageing process (Figure 5a,b). This result indicates that the molecules of DOB-719 associated in the complex with the SWNTs are more stable towards hydrolysis, whereas the free dye molecules (monomers) degrade over time. This effect is supported by the spectral change in the absorption of the as-prepared and the aged mixtures of DOB-719 with SWNTs (Figure 3a, curves 3 and 4) showing a higher stability for the peak at λ_abs_ =742 nm of the complexes than the monomeric peak at λ_abs_ = 692 nm (Figure 3a, curves 4).

Thus, the mechanism of PL quenching for DOB-719 and the SWNTs is similar to the astraphloxin system [12], as the quenching of both dyes in the mixtures is approximately the same. Particularly, comparing our systems of SWNTs–surfactant–dye with the system of SWNTs–porphyrin without surfactant, which demonstrated much stronger quenching (ca. 1000 times) [21]. Therefore, in the SWNTs–SDBS–DOB-719 complexes the surfactant molecules could remain between the dye and the nanotubes. However, the direct contact of the DOB-719 with the SWNTs is also possible. In fact, the distance between DOB-719 and the SWNTs has to be much smaller (or overlap of their π-conjugated systems is stronger) than between the SWNTs and astraphloxin [12]. This statement is supported by a much stronger redshift of the E_11_ peak (22–40 meV vs 6 meV [12]), evident quenching of the intrinsic PL emission of the SWNTs, and no interaction of the dye with SDBS in the complexes of SWNT–SDBS–DOB-719 in comparison with the system of SWNT–SDBS–astraphloxin [12,19].

## Conclusion

We have studied the interaction of the organic dye DOB-719 with SWNTs dispersed by an anionic surfactant (SDBS) in water using absorption and PL spectroscopy. We have found that DOB-719 is associated with the SWNTs and forms nano-structured complexes via π–π stacking by the hydrophobic conjugated frame facing the aqueous medium with the hydrophilic groups SO_3_^–^ and COO^–^. The interaction is evidenced by new optical features in the range of the intrinsic excitation of the dye because of resonant energy transfer from the dye to the SWNTs. In the mixtures of DOB-719 with SWNTs, a new absorption peak corresponding to the dye molecules associated to the SWNTs has emerged being 50 nm redshifted in comparison to the monomeric peak of neat DOB-719 absorption. The new PL peaks featuring the RET have appeared at the excitation wavelength 735 nm and the emission wavelength corresponding to SWNT E_11_ emission levels. Moreover, the λ_EM_ of PL peaks for the mixtures are strongly redshifted (up to 40 nm) in comparison with the peaks of the neat nanotubes for all the SWNT chiralities evidencing strong dielectric screening of the formed complexes. The new peak developing at the excitation wavelength 735 nm can be used for PL detection of SWNTs in aqueous environment. In the mixture of DOB-719 and SWNTs, the monomeric dye molecules were either in free form and degraded severely, or associated in a complex with the SWNT micelles and became more stable. Thus, the interaction between DOB-719 and the SWNT opens a new way to design efficient and tailorable optical probes for not only sensitivity, but also selectivity of the PL detection towards the nanotube diameters.

## Experimental

### Sample preparation

#### Synthesis DOB-719

**Scheme 1:**
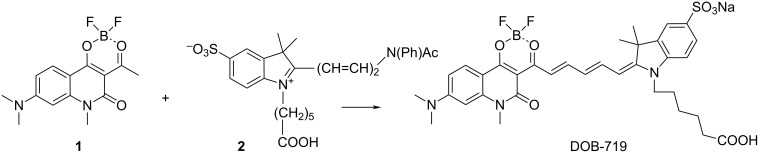
Synthesis of DOB-719.

DOB-719 was synthesised as shown in Scheme 1 by the reaction of (2 mmol) dioxaborine **1** [13] with (2.2 mmol) of hemicyanine **2** [26] and (2.2 mmol) of triethylamine (TEA). The resultant mixture was stirred at room temperature in 6 mL of pyridine for 3 h. After vacuum distillation of the reaction mixture, the residue was grinded with isopropyl alcohol (iPrOH) and filtered out. The product was dissolved in 80% aqueous ethanol and then the solution of sodium acetate (0.2 g) in ethanol was added. The precipitate was filtered out and purified via chromatography on a silica column (7:3 v/v acetone/methanol as an eluent) to yield 300 mg (20%) of product as a green powder. ^1^H NMR (300 MHz, DMSO-*d*_6_) δ 1.3–1.36 (m, 2H, CH_2_), 1.54–1.63 (m, 10H, 2CH_3_+2CH_2_), 2.09–2.13 (m, 2H, CH_2_), 3.14 (s, 6H, N(CH_3_)_2_), 3.54 (s, 3H, NCH_3_), 3.94–3.98 (m, 2H, CH_2_), 6.19 (d, *J* = 15 Hz, 1H), 6.34 (s, 1H), 6.52 (t, *J* = 15 Hz, 1H), 6.78 (d, *J* = 9 Hz, 1H), 7.19 (d, *J* = 9 Hz, 1H), 7.58 (d, *J* = 6 Hz), 7.68–7.73 (m, 2H), 7.87 (d, *J* = 9 Hz, 1H), 8.13 (t, *J* = 12 Hz, 1H), 8.29 (t, *J* = 12 Hz, 1H).

#### Preparation of SWNTs

SWNTs were prepared in a similar manner as described in [12], where purified SWNTs (CoMoCAT) were used to prepare the SWNT dispersions. 1.2 mg of the SWNTs were dispersed in 20 mL of deionized (DI) water in the presence of 6.5 mg sodium dodecylbenzene sulfonate (SDBS). We selected SDBS among other ionic surfactants because of its high efficiency in dispersing SWNTs [27–28]. The above dispersions were exposed to ultrasonication (NanoRuptor, Diagenode) for 1 h at 21 kHz and 250 W. Then, the dispersions were subjected to ultracentrifugation for 3 h at 17 °C at 45000 rpm (Beckman Coulter Optima Max-XP, MLS 50 rotor) to remove the aggregate phase and obtain the supernatant solutions of debundled SWNTs. The top 70% of the dispersion is then decanted. To prepare mixture of DOB-719, we used 20% of the initial SWNT dispersion with SDBS to maintain the dye admixture with the SWNTs at the premicellar concentration (0.065 mg/mL). The origin and level of purity for all materials used in this manuscript are described in Supporting Information File 1.

### Experimental setup

The absorption spectra in the visible and NIR ranges were measured using a Lambda 1050 UV–vis–NIR (Perkin Elmer) spectrometer with 1.5 nm increment. The deionized water was used as a reference in all measurements of the absorption spectra. The PL emission spectra at various excitation wavelengths were recorded using a Horiba NanoLog excitation–emission spectrofluorometer equipped with a InGaAs array detector cooled by liquid nitrogen and Si detector to produce PL excitation–emission maps (PLE maps), with the *X*-axis representing the emission wavelength (λ_EM_) and the *Y*-axis representing the excitation wavelength (λ_EX_). The PL measurements in the NIR were performed using entrance/exit slits of 14 nm in width for both the excitation and emission monochromators. Entrance/exit of 2 nm slits were used for both the excitation and emission monochromators in the visible range measurements. All samples in this manuscript were measured under standard laboratory conditions. Also, the pH value was maintained at 7 to ensure a good dispersion of SWNTs and avoid bundling [29].

## Supporting Information

File 1 Additional figures and discussion.
