# Supplementary material for: A dioxaborine cyanine dye as a photoluminescence probe for sensing carbon nanotubes
Source: Beilstein J Nanotechnol. 2016 Dec 14;7:1991–9. doi: 10.3762/bjnano.7.190 (PMC5238636; doi:10.3762/bjnano.7.190)
Supplement: File 1 — Additional figures and discussion. [file Beilstein_J_Nanotechnol-07-1991-s001.pdf]

Supporting Information

for

A dioxaborine cyanine dye as a photoluminescence probe for sensing carbon nanotubes

Mohammed Al Araimi*[1,2], Petro Lutsyk[1,3], Anatoly Verbitsky[3], Yuri Piryatinski[3], Mykola Shandura[4] and Aleksey Rozhin*[2]

Address: [1]Nanotechnology Research Group, Aston Institute of Photonic Technologies, School of Engineering & Applied Science, Aston University, Aston Triangle, B4 7ET Birmingham, UK; [2]Engineering Department, Al Musanna College of Technology, Muladdah Musanna, P.O. Box 191, P.C. 314, Sultanate of Oman; [3]Institute of Physics, National Academy of Sciences of Ukraine, 46, prospekt Nauky, 03680 Kyiv, Ukraine and [4]Institute of Organic Chemistry, National Academy of Sciences of Ukraine, 5, Murmanska str., 02660 Kyiv, Ukraine

Email: Aleksey Rozhin - a.rozhin@aston.ac.uk; Mohammed AlAraimi - alarammm@aston.ac.uk

* Corresponding author

Additional figures and discussion

The DOB-719 dye was synthesised at the Institute of Organic Chemistry, National Academy of Sciences of Ukraine. The DOB-719 purity was determined using NMR as well as liquid chromatography and mass spectrometry (LC-MS) and found to be approx. 95%. According to SWeNT Inc. technical data sheet, SWNT powder (purchased from SWeNT Inc., CG100, Lot # 000-0012) has a carbon purity of 90 wt % and non-carbon content equal to 10 wt %. The sodium dodecylbenzene sulfonate (SDBS) purchased from (Sigma-Aldrich #289957) is technical grade material (approx. 90%). According to the specification sheet, this material might contain positional isomers, isomers of different chain lengths or branched isomers, but the infrared and NMR spectra of the material conform to the SDBS structure.

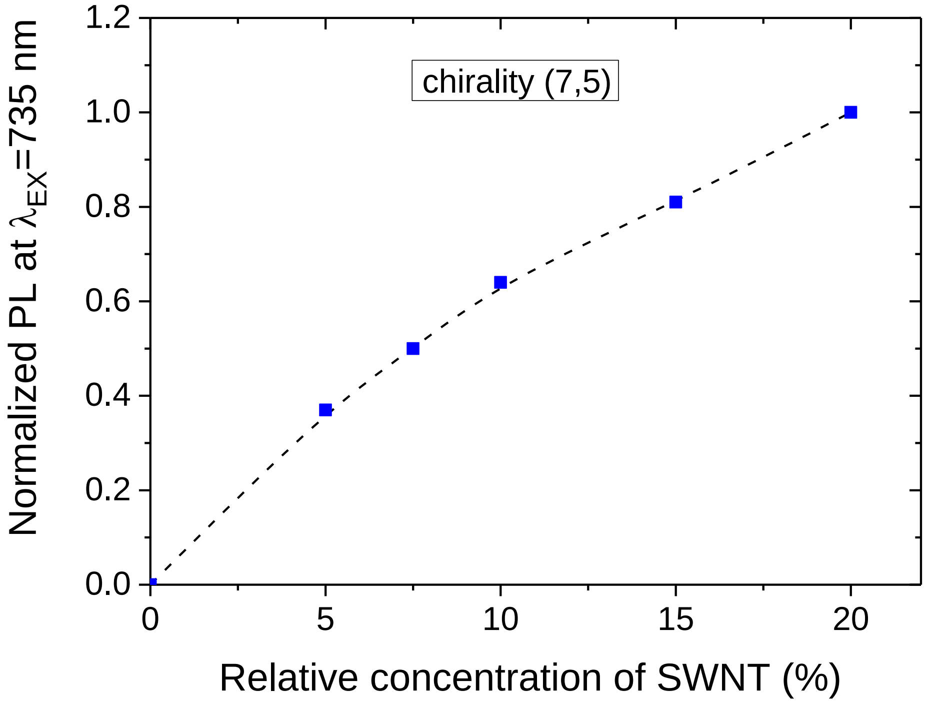

Figure S1: The SWNT concentration dependence for normalized intensity of the PL at λ_{EX} = 735 nm and λ_{EM} = 1059 corresponding to (7,5) chirality emission. 100% of the relative concentration of SWNT corresponds to the initial dispersion of the SWNT, where the dilutions were used to prepare the mixtures of DOB-719 with lower concentrations of SWNT. The concentration of DOB-719 is 0.001 mg/mL in all the samples.

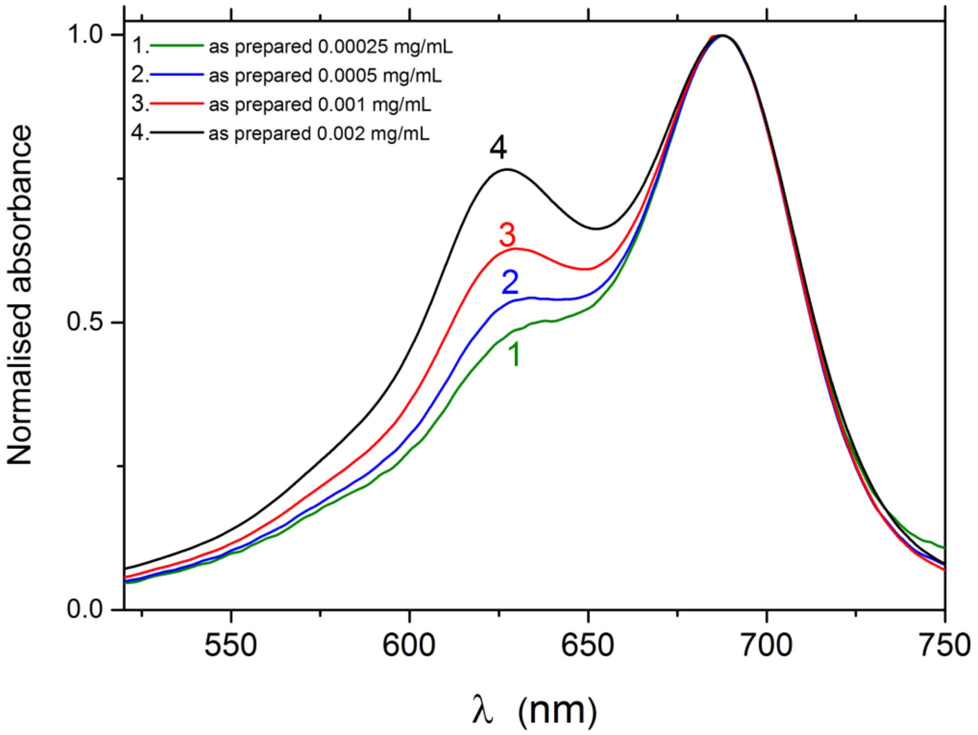

Figure S2: Normalized absorption spectra of the as prepared aqueous solution of DOB-719 at the concentrations of 0.00025 mg/mL (1), 0.0005 mg/mL (2), 0.001 mg/mL (3), and 0.002 mg/mL (4).

In Figure S2, absorption spectra of DOB-719 in water characterise the presence of monomeric form of the dye, where the absorption peak at λ = 687 nm is belonging to monomeric form of the dye and does not shift irrespective of the dye concentration with good agreement to [1]. Moreover, based on Gaussian deconvolution of the low concentration spectrum (0.00025 mg/mL; Figure S2, curve 1) we have estimated that the second vibrational transition of the dye monomers has a band with maximum at approximately λ = 631 nm (1.96 eV). According to [1], an increase of the concentration of the DOB-719 dye in aqueous solution above 0.001 mg/mL results in growing contribution of DOB-719 dimers (two dye monomers attached together) featured by a blue-shifted peak. A growth of the second maximum in the spectral range of 600–650 nm occurs at concentrations higher than 0.00025 mg/mL evidencing formation of DOB-719 dimers. The dimers start to form substantially at the concentrations higher than 0.00025 mg/mL resulting in growth of the second maximum in the spectral range of 600–650 nm. The Gaussian deconvolution of

absorption spectra at high concentrations (Figure S2, curves 2–4) shows that the dimeric peak of absorption is positioned at approximately 623 nm (1.99 eV).

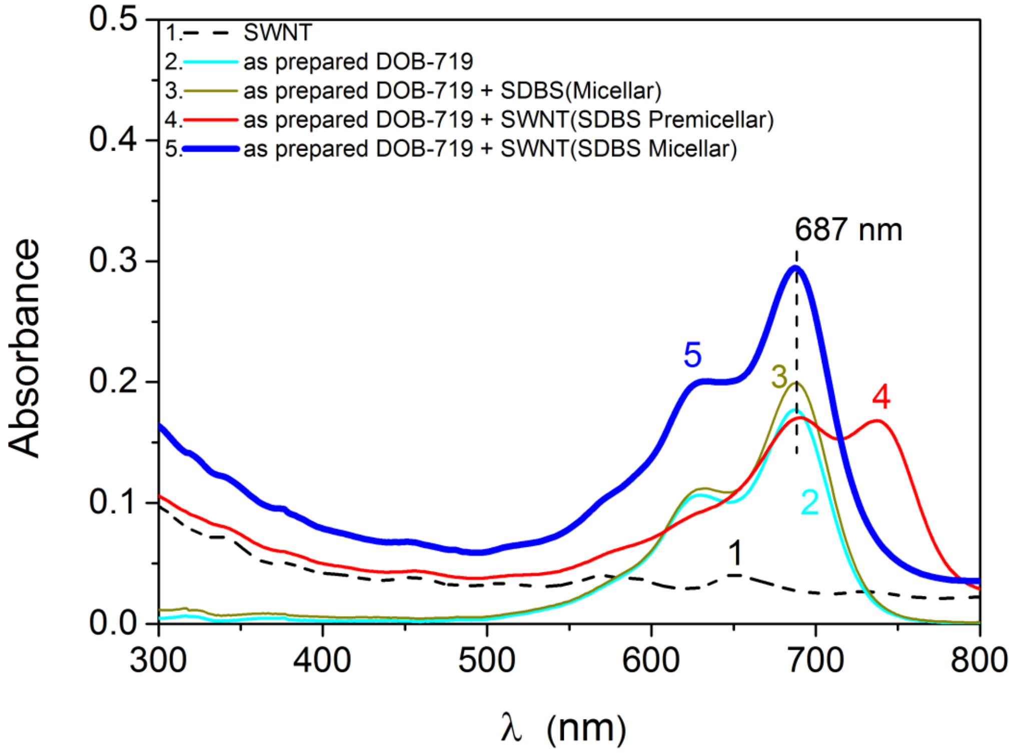

Figure S3: Absorption spectra of neat SWNT dispersions (1), solutions of neat DOB-719 (2), the mixtures of as prepared DOB-719 with SDBS at micellar concentration of 0.4 mg/mL (3), the mixtures of as prepared DOB-719 and SWNT with SDBS at premicellar concentration of 0.065 mg/mL (4), and mixtures of DOB-719 and SWNT with SDBS at micellar concentration of 0.4 mg/mL (5). The concentration of DOB-719 is 0.001 mg/mL.

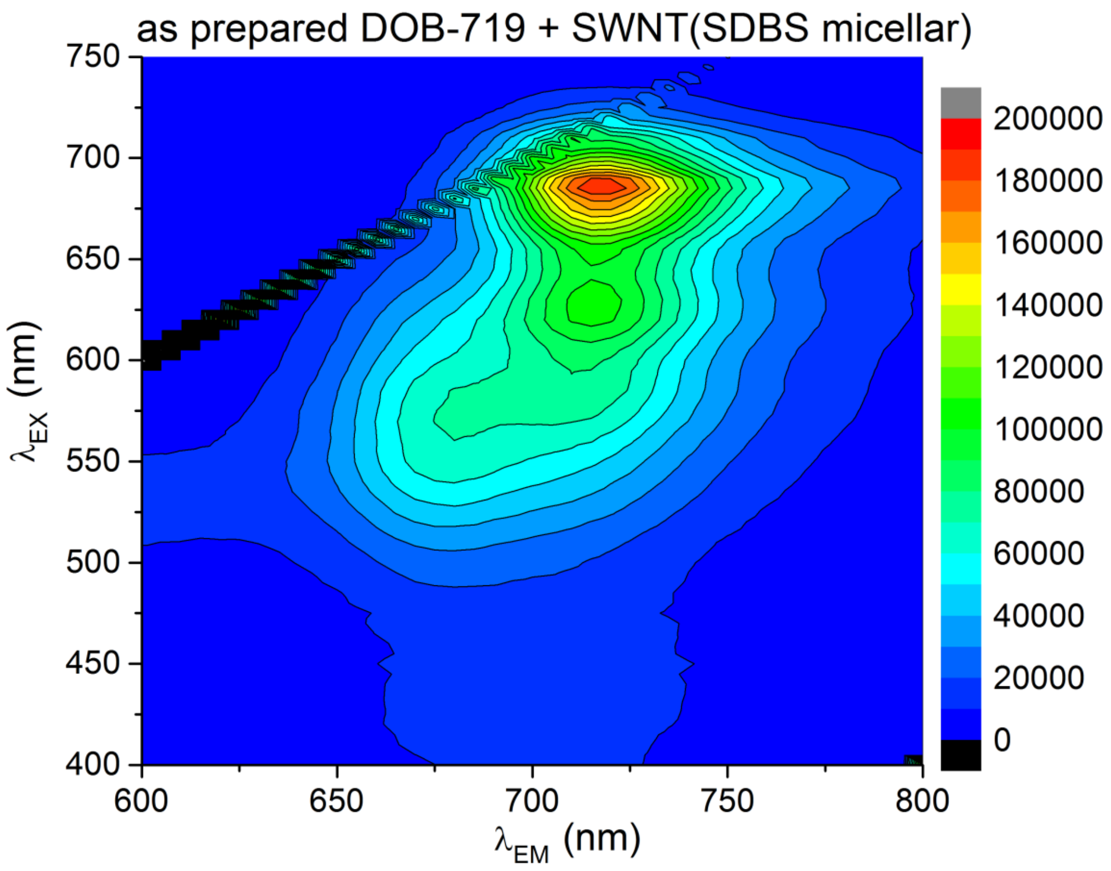

Figure S4: PLE map in visible range for the as prepared mixtures in water of DOB-719 and the SWNT with SDBS at micellar concentration of 0.4 mg/mL. High PL intensities are coded in red colour, whereas low intensities are coded in blue colour. The concentration of DOB-719 is 0.001mg/mL.

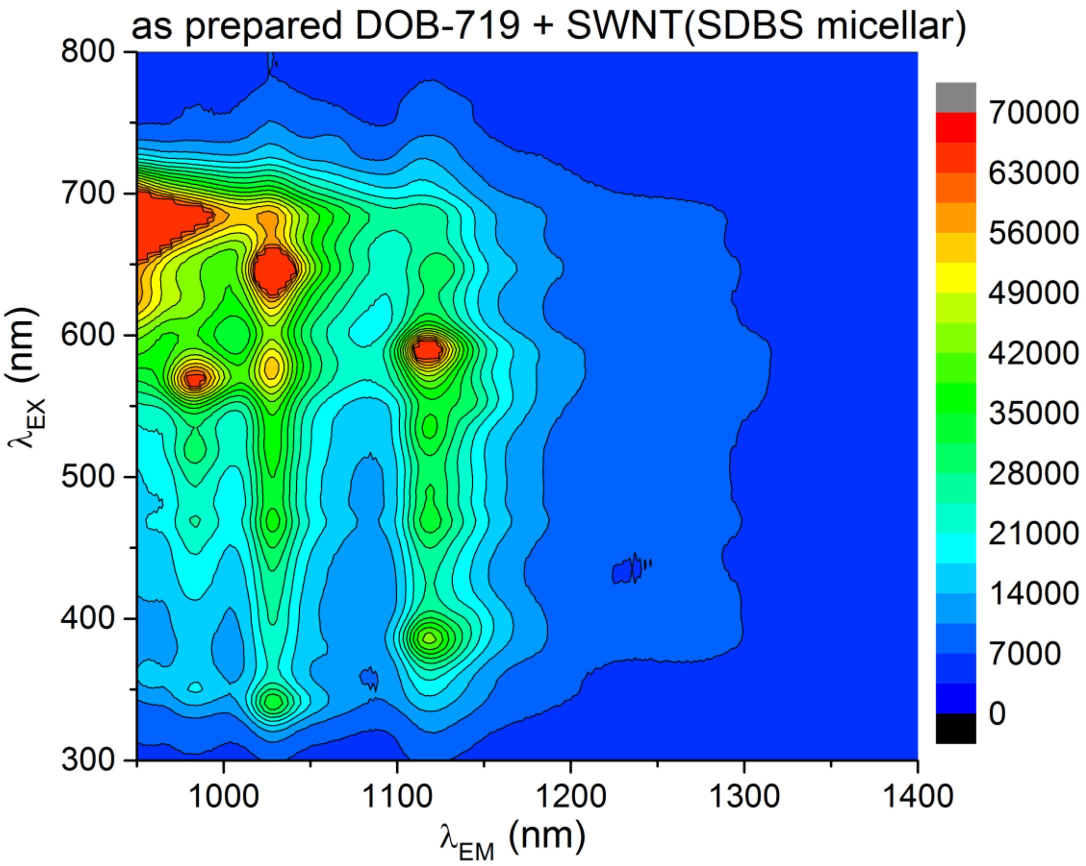

Figure S5: PLE map in NIR range for the as prepared mixtures in water of DOB-719 and the SWNT with SDBS at micellar concentration of 0.4 mg/mL. High PL intensities are coded in red colour, whereas low intensities are coded in blue colour. The concentration of DOB-719 is 0.001mg/mL.

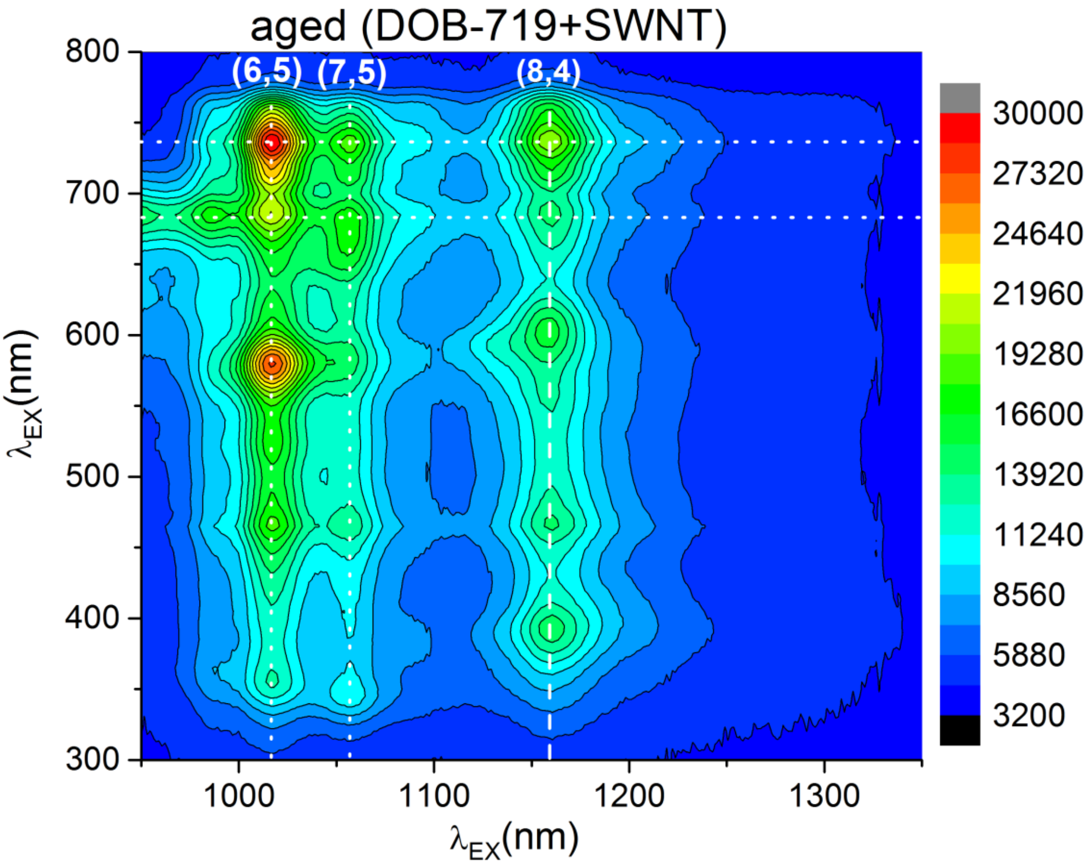

Figure S6: Aged mixture of DOB-719 with SWNT. Vertical dashed lines indicate λ_{EM} positions of E_{11} for SWNT (6,5), (7,5), and (8,4) chiralities, horizontal dashed lines indicate the position of new PL peaks at λ_{EX} = 735 nm (the RET from DOB-719 to the SWNT) and the position of DOB-719 monomers maximum at λ_{EX} = 685 nm. High PL intensities are coded in red colour, whereas low intensities are coded in blue colour. The concentration of DOB-719 is 0.001 mg/mL.

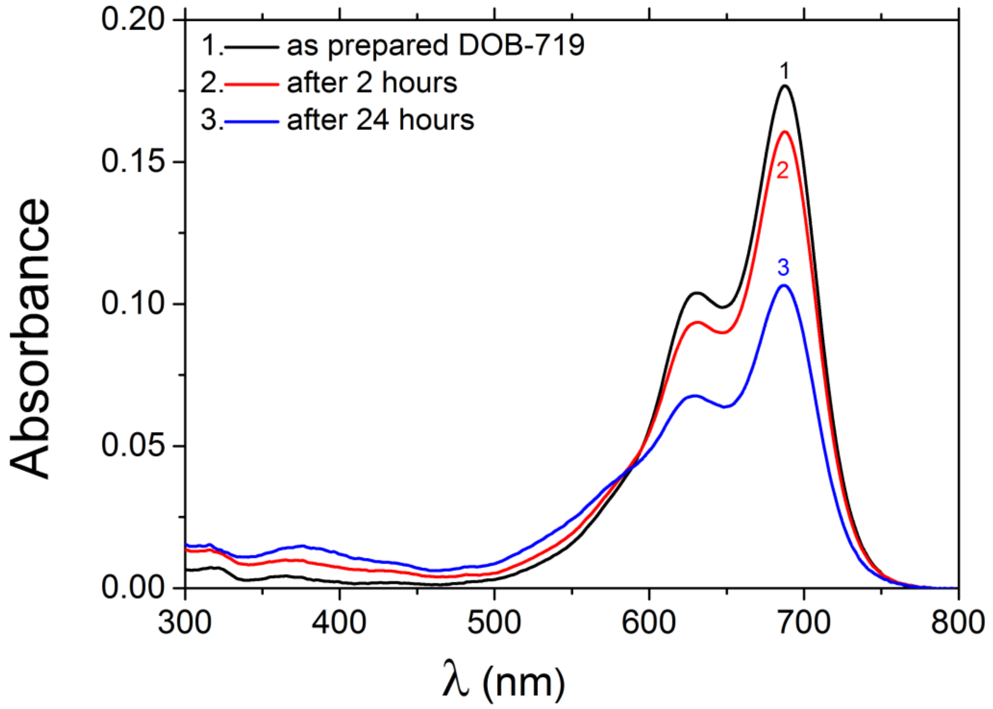

Figure S7: Absorption spectra for aqueous solutions of neat DOB-719 as prepared (1), after 2 h (2) and after 24 h (refers to the aged samples) (3). The concentration of DOB-719 is 0.001 mg/mL.

As shown in Figure S7, we have studied the absorption of neat DOB-719 in water and observed some spectral changes within 24 h. The absorption intensity of the dye decreases in the range of first electronic transition (600–720 nm) and increases in the range below 600 nm. Particularly, new bands start to develop in the ranges of 335–460 nm and 500–600 nm. The decrease of the intensity for the dye monomer and growth of new bands can be explained due to the degradation of dioxaborine dye, particularly hydrolysis of dioxaborine cycle, where the basic mechanism of the hydrolysis is the removal of BF_2-group [2].

References

1 Shandura, M.; Kovtun, Y. P.; Yakubovskyi, V.; Piryatinski, Y. P.; Lutsyk, P.; Perminov, R.; Arif, R.; Verbitsky, A.; Rozhin, A. *Sens. Lett.* **2014,** *12,* 1361–1367. doi:10.1166/sl.2014.3315

2 Gerasov, A. O.; Zyabrev, K. V.; Shandura, M. P.; Kovtun, Y. P. *Dyes Pigm.* **2011,** *89,* 76–85. doi:10.1016/j.dyepig.2010.09.007
